# Post-industrial context of cassava bagasse and trend of studies towards a sustainable industry: A scoping review – Part I

**DOI:** 10.12688/f1000research.110429.1

**Published:** 2022-05-23

**Authors:** José Gabriel Serpa-Fajardo, Elvis Judith Hernández-Ramos, Gregorio Fernández-Lambert, Luis Carlos Sandoval-Herazo, Ricardo David Andrade-Pizarro

**Affiliations:** 1 Tecnológico Nacional de México-Campus Misantla, Misantla, Veracruz, 93821, Mexico; 2 Departamento de Ingeniería Agroindustrial, Universidad de Sucre, Sincelejo, Sucre, 700001, Colombia; 3 Facultad de Ingenierías, Departamento de Ingeniería de Alimentos, Universidad de Córdoba, Montería, Córdoba, 230002, Colombia

**Keywords:** waste bioconversion, emerging technologies, biomass, industrial drying

## Abstract

**Background:** The cassava starch industry is recognized as a source of negative externalities caused by the agroindustrial waste ‘cassava bagasse’. Even though options for bioconversion of cassava bagasse have been introduced, it is also true that hundreds of tons of this waste are produced annually with the consequent negative environmental impact. This agroindustrial context highlights the need for further research in technological proposals aimed at lowering the water contained in cassava bagasse.

**Methods:** We report a scoping review of studies from 2010–2021 that mention the uses of cassava bagasse, as well as the technological options that have become effective for drying fruits and vegetables. The method used for selecting articles was based on the Preferred Reporting Items for Systematic Review and Meta-Analyses extension for Scoping Reviews (PRISMA-ScR) method. Articles selected were taken from the databases of ScienceDirect, Google Scholar, Scopus and Springer.

**Results** **:** This review highlights fruit and vegetable osmotic dehydration and drying studies assisted by the combination of emerging technologies of osmotic pressure, ultrasound, and electrical pulses. Studies that take advantage of cassava bagasse have focused on biotechnological products, animal and human food industry, and development of biofilms and biomaterials.

**Conclusions: **In this review, we found 60 studies out of 124 that show the advantages of the residual components of cassava bagasse for the development of new products. These studies do not mention any potential use of bagasse fiber for post-industrial purposes, leaving this end products’ final use/disposal unaddressed. A viable solution is osmotic dehydration and drying assisted with electrical pulse and ultrasound that have been shown to improve the drying efficiency of fruits, vegetables and tubers. This greatly improves the drying efficiency of agro-industrial residues such as husks and bagasse, which in turn, directly impacts its post-industrial use.

## Introduction

Cassava crop ( *Manihot esculenta Crantz*) has had significant growth in agriculture worldwide, particularly in the tropical countries of Latin America, the Caribbean, Asia, Africa, and Oceania. Because of its tolerance to drought, the ability to thrive in marginal soils, and flexibility for planting and harvesting, cassava is currently considered a multipurpose crop responding to food and commercial expectations of emerging countries ( Howeler *et al*., 2013 cited in Zainuddin *et al*., 2017).

Annual world production of cassava is approximately 303.5 million tons ( FAO, 2019) which, once harvested, shows the great constraint of being a highly perishable product. For this reason, besides being part of the food diet, the harvest surplus is processed into chips, pellets, flour, bioethanol, cookies, balanced feed for animals, as well as for producing native starch and modified starches.

Starch production from cassava is one of the industrial processes of recent global importance, especially in the textile, chemical, pharmaceutical, food, and beverage industries, even in gastronomy, powdered beverages, and others. The FAO (2019) reported that the world market for cassava starch reached an approximate production of eight million tons in 2018, with a compound annual rate of 4.73% between 2011 and 2018. Thus, for 2024 it is expected to exceed 10 million tons, with a compound annual rate of 3.76% from 2019 to 2024.

Obtaining industrial starch from cassava generates a high organic load of cassava bagasse, with environmental impact on the soil, water, and air, requiring an alternative treatment for its reincorporation into the environment. Processing 250–300 tons of cassava tubers results in approximately 1.6 tons of solid peel and approximately 280 tons of cassava bagasse with high moisture content ( Escaramboni *et al*., 2018; Florencia *et al*., 2019). Zhang *et al*. (2016) report that for every ton of processed cassava, between 0.93 and 1.12 tons of bagasse and cassava peel are produced, which is later discarded, and in the best of cases disposed of in sanitary landfills technically in the open without any treatment, negatively impacting the environment ( John, 2009; Grande Tovar, 2016; Morales *et al*., 2018; Oyewole and Eforuoku, 2019; Grasso, 2020).

Cassava bagasse is a fibrous material with a humidity percentage higher than 85%, difficult to efficiently remove by conventional drying. This by-product contains relatively low content of proteins, ashes, and fats, and approximately 16% dry matter, mainly made up of carbohydrates with up to 82.85% with a content greater than 50% starch ( Polachini *et al*., 2016; De Souza *et al*., 2018; Travalini *et al*., 2019), and fiber between 15–50% ( Chen *et al*., 2015). With all this, cassava bagasse is not being used by the cassava starch production industry, generating daily large volumes, fermenting in an uncontrolled way, and producing unpleasant odors. This waste has become an issue of environmental pollution for the same factories and neighboring regions ( Grasso, 2020).

Efforts to use alternative low-cost and renewable materials as raw materials to generate new products at an efficient cost and environmentally friendly, have led to implementation of innovative technologies to use agroindustrial waste to obtain valuable, useful, and beneficial products in other industrial processes ( Sharmila *et al*., 2020). Nonetheless, generation of agroindustrial waste in the varied industrialization stages is currently a problem with negative environmental impacts worldwide because in most cases they are not properly processed or handled ( Vargas & Pérez, 2018). The main problem found during waste management with high humidity content such as cassava bagasse, is the low use by other industrial sectors due to issues in handling, storage, transport, and conservation. This requires them to be subjected to a drying process ( Polachini *et al*., 2016) or to humidity elimination to lower their volume or mass to favor handling and subsequent use in different industrial processes.

Different alternatives for drying have been abundantly used in the agroindustrial sector, among which, solar drying, hot air drying, spray drying, or osmosis dehydration stand out ( Vargas & Pérez, 2018) among other emerging technological options such as electric pulses, high hydrostatic pressure, ultrasound, centrifugal force, vacuum, gamma irradiation, and traditional technologies. All of which have been shown to improve the efficiency of osmotic dehydration and drying operations to reduce drying time with lower energy cost ( Ahmed *et al*., 2016).

These combined techniques of emerging and conventional technologies have been efficiently applied in the osmotic dehydration and drying processes of various products, such as fruits, vegetables, fillets, and tubers (yams, cassava, potatoes, and sweet potatoes), as well as in some polymeric matrices. These results make it interesting to study its impact on the efficiency of the osmotic dehydration process and subsequent drying of the cassava bagasse, as well as other agroindustrial waste, especially using emerging technologies that are attractive due to their simplicity and economy for these processes. The lack of studies on efficient techniques for drying agroindustrial waste opens up the need to advance in technological innovation processes to identify opportunities for the economic revaluation of the agroindustry and its wastes. There is a growing interest in finding technological alternatives to provide added value to these agroindustrial waste with high moisture content ( Florencia *et al*., 2019).

In order to guide the cassava starch industry towards a sustainable agribusiness, this article synthesizes the technological advances reported in scientific literature from 2010–2021 for the use of cassava bagasse, as well as the technological options that have become good solutions in the dehydration of fruits, tubers, and vegetables; thus, proposing the hypothesis that they can be adapted to cassava bagasse drying. Derived from this technological synthesis, this article contributes to the knowledge of the industrial and scientific community. Recommendations of technological alternatives for the industrial scaling of options for the use of cassava bagasse are proposed. These include implementation of efficient drying processes, particularly through application of combined techniques of emerging and traditional technologies in the field of post-agroindustrial drying of cassava bagasse and other similar agroindustrial residues.

To this end, the following sections of this article describe the methodological approach of this research, order of cassava world production, its industrialization and studies on the use of cassava bagasse. Emerging technologies are also described, which apply good results in the field of fruit, vegetable, and tuber waste. These can be replicated as options to the drying of cassava bagasse to minimize the negative externality of this waste and manage its efficient handling in post-industrial processes as a by-product of high industrial value.

## Methods

For the systematic review of the scientific literature, according to the objectives of study, the following databases were used: ScienceDirect, Google Scholar, Scopus and Springer. The Preferred Reporting Items for Systematic Review and Meta-Analyses extension for Scoping Reviews (PRISMA-ScR) methodology was used for identification, selection, eligibility and inclusion, results synthesis, and discussion ( Tricco *et al*., 2018), and a PRISMA 2020 flow diagram was created ( Neal *et al.*, 2021). This study is reported in line with the PRISMA guidelines for scoping reviews ( Serpa-Fajardo *et al*., 2022).

The annual world production of cassava bagasse was estimated based on the statistics from the Food and Agriculture Organization ( FAO, 2019), while the conversion factors for the generation of this residue were taken from what was reported in the scientific literature.

### Eligibility criteria

Only records corresponding to the publication period between 2010 and 2021 were considered, and only reviews articles and research articles were examined. Furthermore, only reviews and research articles addressing the current cassava bagasse issue and its different use options were considered, excluding articles on cassava, use of other cassava residues, cassava flour and cassava starch.

Reviews and research articles that addressed the combined techniques of osmotic dehydration as pretreatment to improve the efficiency of the convective air-drying process of agro-industrial products were also considered, and those that employ the support of emerging ultrasound or electrical pulse technologies in the osmotic dehydration and drying processes to further improve their efficiency. Articles that address other types of emerging technologies different to ultrasound and electrical pulses, in addition to articles that address these technologies individually and not as combined techniques were excluded, e.g., articles addressing only osmotic dehydration operation or only the drying operation.

### Search strategy

The most recent search date was November 2021. The keywords used in the search were: ‘ *cassava bagasse*’; ‘ *cassava waste assessment*’; ‘ *use of cassava bagasse’.* The title, abstract and keywords of the articles were searched, chained as follows: *‘cassava bagasse’* OR *bran* OR *cassava residues* AND *valuation* OR *utilization* AND NOT *starch.* Similarly, for the purposes of reviewing the application of combined techniques of emerging ultrasound and electrical pulse technologies with osmotic dehydration operations and conventional drying of various products, the following keywords were used: ‘ *drying’*; ‘ *osmotic dehydration*’, ‘ *emerging technology’*, ‘ *ultrasound’* and ‘ *electrical pulses*’. The title, abstract and keywords of the articleswere searched as follows: *osmotic dehydration* AND *drying*; *ultrasound* AND *dehydration* AND *drying*; *Electrical pulses* AND *osmotic dehydration* AND *drying.*

### Selection of records and data management

Record selection was carried out by two authors: J.G. Serpa-Fajardo and E.J. Hernández-Ramos, independently, based on the predetermined inclusion and exclusion criteria previously mentioned. Discrepancies were resolved through discussion.

Given the particular features of the records selected in this scoping review, such as the heterogeneous evidence base, data management was performed using thematic and content analysis according to the objectives of this study, grouping results in common applications as follows: studies related to the use of cassava bagasse were grouped by publication year, country conducting the study and according to the addressed application field. Consistently, research work related to the application of the combined techniques were grouped according to the publication year, countries that carried out the study and according to the combination of techniques used. Processing, illustration and synthesis of results were developed in a narrative format, table and figure, through the use of Microsoft Excel (version 16.0) spreadsheet software (RRID:SCR_016137).

## Results

In this review carried out to establish the current problems of cassava bagasse and its different use options according to the adopted search strategy, an initial record of 5,215 results was obtained: ScienceDirect (1,260), Google Scholar (3,020), Scopus (128), and Springer (807). Meanwhile to establish the current application state of combined techniques of emerging technologies of ultrasound and electrical pulses with the operation of osmotic dehydration and conventional drying of various products, based on the mentioned search strategy, a record of initial results obtained was of 15,973 documents: ScienceDirect (8,534), Google Scholar (5,180), Scopus (2,019), and Springer (240).

**Figure 1.**
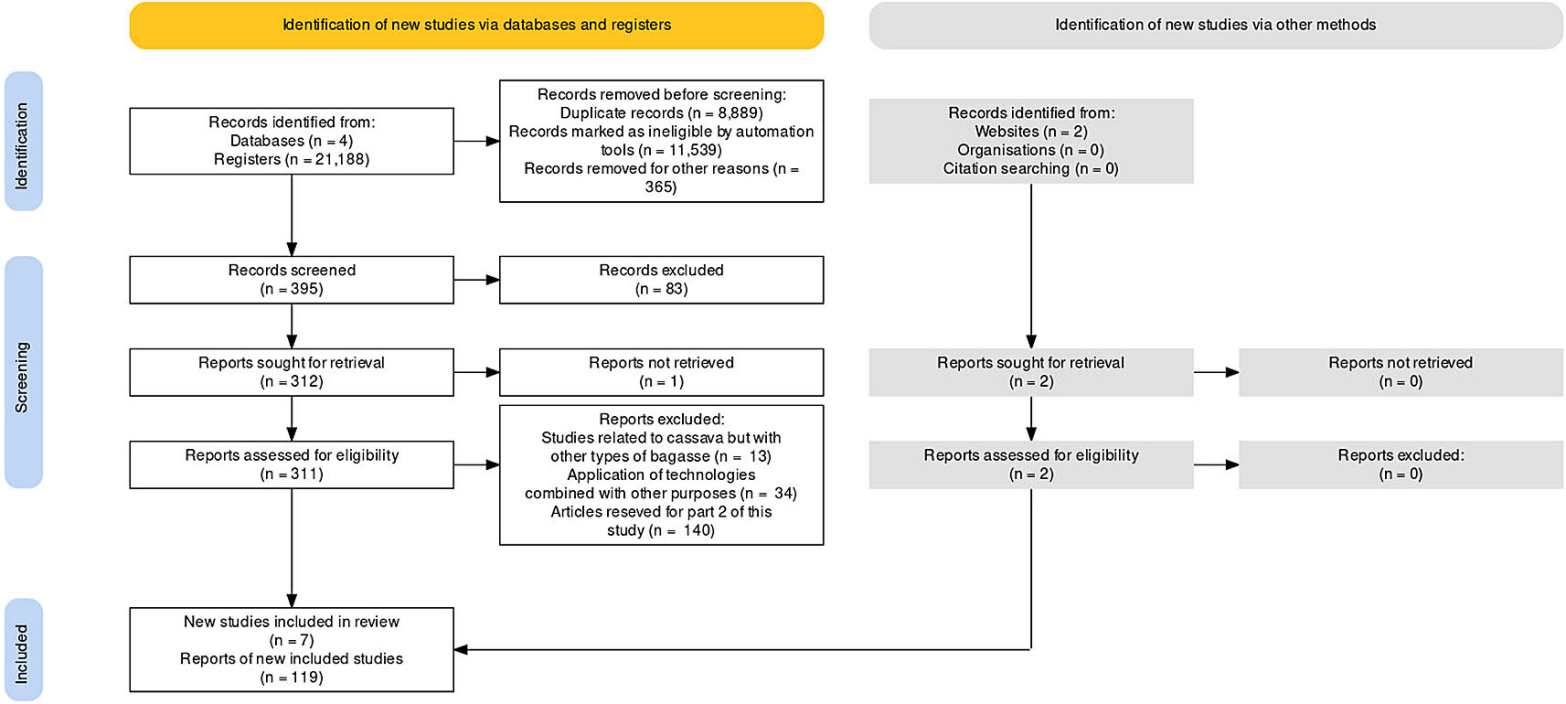
PRISMA flow diagram: selection of sources of evidence.

Starting from the total number of records (21,188 total documents), the screening process was conducted according to the study objectives. First, of the records were restricted by publication year (2010–2021) and only reviews and research articles were selected. A total of 20,793 documents were discarded including repeated articles and articles regarding other areas not included in this review ( Figure 1). Out of the 395 articles that remained an additional 83 articles were excluded, namely: 21 articles on cassava use, use of other cassava residues, cassava flour, cassava starch and 62 articles that addressed other types of emerging technologies other than ultrasound and electrical pulses, as well as articles that address these technologies individually and not as combined techniques, e.g., articles that addressed only osmotic dehydration or the drying operation. Only articles that addressed the combined techniques of osmotic dehydration as a pretreatment to improve the efficiency of the convective air-drying process of agro-industrial products, and those that employ emerging ultrasound or electrical pulse technologies in osmotic dehydration and drying processes to improve efficiency were selected. Moreover, we excluded one publication that was not retrievable, 13 articles corresponding to studies related to cassava but with other types of bagasse, 34 articles that were discarded for applying said technologies combined with other purposes, and 140 articles addressed in Part II of this study. Part II reports the current status of osmotic dehydration technology, used alone or assisted by emerging ultrasound technologies or electrical pulse technology, which today are used to improve drying efficiency in various agro-industrial products; potentially useful findings so that these combined drying techniques are addressed through new research aimed at achieving an efficient process of water removal in cassava bagasse and other agroindustrial residues.

Finally, a total of 124 articles were selected: seven review articles and 117 research articles, of which 60 corresponded to studies on the use of cassava bagasse, 44 were about the use of the combined techniques of ultrasound, electrical pulses, osmotic dehydration, and drying, and 20 about the generalities of the cassava industry related to the assessment of cassava bagasse. Futhermore, two FAO reports from 2019 and 2020 were considered.

It was established that the cultivation of cassava ( *Manihot esculenta, crantz*) has had significant growth in world agriculture ( Lobell *et al*., 2008 and Howeler *et al*., 2013, cited in Zainuddin *et al*., 2017), and today it is considered the sixth most important crop in the world after wheat, rice, corn, potato, and barley ( Otekunrin & Sawicka, 2019; Adepoju & Oyewole, 2013, cited in Dada, 2018). Cassava is ranked as the fifth most used starch source in the world and the third among the food sources consumed in tropical regions ( Florencia *et al.,* 2019), which has contributed to the global expansion of rapid commercialization of this crop and large-scale investments in order to expand product processing ( OECD y FAO, 2020). Table 1 highlights that ten countries hold 74.4% of the world production of cassava, where Nigeria, the Democratic Republic of Congo, Thailand, Ghana, and Brazil contribute 56.1% to world production.

**Table 1.** Main producing countries of cassava.

No.	Country	Production (Million tons/year)	Participation (%)
1	Nigeria	59.19	19.5
2	Decomocratic Republic of Congo	40.05	13.2
3	Thailand	31.07	10.2
4	Ghana	22.44	7.4
5	Brazil	17.49	5.8
6	Indonesia	14.58	4.8
7	Cambodia	13.73	4.5
8	Vietnam	10.10	3.3
9	Angola	9.00	3.0
10	United Republic of Tanzania	8.18	2.7

In addition to its fresh consumption, cassava bagasse has an agroindustrial use with the highest demand for ethanol production and other biofuels since 2000. Cassava bagasse is also used in the food sector as a raw material to produce traditional foods, to obtain native and modified starches, and in balanced animal feed ( Edhirej *et al*., 2017). Nonetheless, industrialization of cassava generates large amounts of waste rich in organic matter and suspended solids with serious environmental implications in the absence of post-harvest treatments. On the other hand, the complex biochemical composition with high organic content of cassava industrial waste provides great potential for bioconversion into value-added products, thus, providing economic sustainability to the cassava industry ( Zhang *et al*., 2016; Li *et al*., 2017).

The most important postharvest constraint in cassava cultivation is its short shelf life. Therefore, one of the best options to increase the value of harvested cassava to benefit rural economies is to process excess cassava for starch production ( Teixeira *et al.*, 2009, cited in Travalini *et al*., 2019). Approximately 60 million tons of starch are extracted annually worldwide from different sources for use in a large number of products. Out of this quantity, eight million tons, equal to 13.3% of the starch produced worldwide, is produced from cassava ( FAO, 2019). Furthermore, cassava and its waste generated by its industrialization have attracted researchers to explore its capacity as a reinforcement material in biodegradable compounds and as a raw material to improve animal and human food safety ( Edhirej *et al*., 2017).

Nonetheless, the high humidity content of some waste from the cassava industry makes it a perishable product in which all kinds of microorganisms proliferate. Decomposition is generated through uncontrolled fermentation causing bad odors and affecting both processing plants as well as the surrounding regions. This has become an environmental problem that must be properly managed.

In this industrial system, Ubalua (2007) highlights that not applying the zero waste principle in this agribusiness can have a negative impact on vegetation and soil, making soils unproductive and devastated due to the biological and chemical reactions taking place among waste, soil and surrounding vegetation. In this sense, the search for applications for cassava waste should contribute to reducing environmental problems ( Leite *et al*., 2017).

Grande Tovar (2016) agree with Escaramboni *et al*. (2018) and Florencia *et al*. (2019) in that processing of 250–300 tons of cassava root produces around 1.16 tons of husk and 280 tons of bagasse. Zhang *et al*. (2016) report that for each ton of cassava processed to obtain 0.25 tons of cassava starch, between 0.93 and 1.12 tons of bagasse and cassava husk are generated, due to imbibition of water. Consequently, considering these conversion factors and the reported annual world production of cassava starch of eight million tons approximately, it is estimated that the approximate annual world generation of cassava bagasse is between 29.76 and 35.84 million tons/year approximately.

This cassava bagasse agroindustrial waste can contain up to 60% by weight of starch on a dry basis, and humidity of about 85%, complicating its short-term storage ( Araujo-Silva *et al*., 2018; María, Martha, & Juan, 2012). The costs associated with handling and disposal of this waste constitutes an undesirable financial burden on the cassava processing industries. As a result of this challenge, cassava processors generally choose to dispose of waste in the open air, which becomes a major environmental issue ( Barros *et al.*, 2012; Olukanni *et al*., 2013; Omilani *et al*., 2015; Cited in Olukanni & Olatunji, 2018).

Table 2 specifically presents the studies on the use of cassava bagasse, classified by author, application field and specific use. Other types of studies are excluded, such as those aimed at use of cassava, cassava leaves and stems, cassava flour, and cassava starch. Cassava studies related to other types of bagasse from other raw materials are also excluded.

**Table 2.** Studies on the use of cassava bagasse.

Author, year	Application	Examples
Liu *et al*. (2018); Ajala *et al*. (2020); De Souza *et al*. (2020); Sujithra *et al*. (2019); Chen *et al*. (2020); Hidayat *et al*. (2018); Pinheiro *et al*. (2018); Godoy *et al*. (2018); Araujo-Silva *et al.* (2018); Sugiharto (2019); Siddhartha *et al*. (2012); Manimala & Murugesan (2017); Trakarnpaiboon *et al*. (2017); Zakariyah *et al*. (2021); Inguillay *et al*. (2021); Gois *et al*. (2020)	Biotechnological Products	Production of fungi, organic acids, xanthan gum, biopigments
Vargas & Pérez (2018); Zanatta *et al*. (2016); Chavadej *et al*. (2019); Huang *et al*. (2019); Escaramboni *et al*. (2018); Ozoegwu *et al*. (2017); Sivamani *et al*. (2018); Martínez *et al*. (2018); Payá *et al*. (2018); Ekop *et al*. (2019); Zhang *et al*. (2016); Xue *et al*. (2017); Albis Arrieta *et al*. (2018); Decarvalho *et al*. (2018)		Manufacture of biofuels (Bioethanol, biomethane, hydrogen, butanol)
Vargas & Pérez (2018); De Souza *et al*. (2018); Salcedo *et al*. (2016); Romero *et al*. (2017); Morales *et al*. (2018); Zheng *et al*. (2020); Rodríguez *et al*. (2016); Fiorda *et al*. (2015); Chiquito *et al*. (2017); Pereira *et al*. (2015); Wali *et al*. (2017); Kaur & Ahluwalia (2017); Bayata (2019); Bizzuti *et al*. (2021)	Animal and human food industry	Substitute for corn in balanced meals, food, cookies, ice cream, etc.
Travalini *et al*. (2019); Ferreira *et al*. (2019); Florencia *et al*. (2019); Edhirej *et al*. (2017); Chen *et al*. (2017); Keller *et al*. (2020); Diabor *et al*. (2019); Silviana *et al*. (2018); López *et al*. (2015); Ribeiro *et al*. (2018); Versino & García (2019); Luchese *et al*. (2018); Leite *et al*. (2017); Coelho *et al*. (2020); Dien & Anh (2021)	Biofilms and Biomaterials	Biodegradable reinforcement material, foams, nanofibers

In this context, the diverse studies aimed at seeking a viable use for cassava bagasse can be ranked into three large areas: 1) Use in the food industry, 2) Biotechnological applications to generate products with greater added value, and 3) Use as a reinforcement material in biomaterials. However, use alternatives reported in these studies leave the results of this research on the use of cassava bagasse at an experimental level, without having the scope of a cost-effective industrial scaling of the said process. In effect, the enduring problem for the industrial scaling of cassava bagasse in these studies is the need to implement innovative and efficient drying processes for this residue, which reduces its mass and volume and eases handling, transport, storage, and conservation.

**Figure 2.**
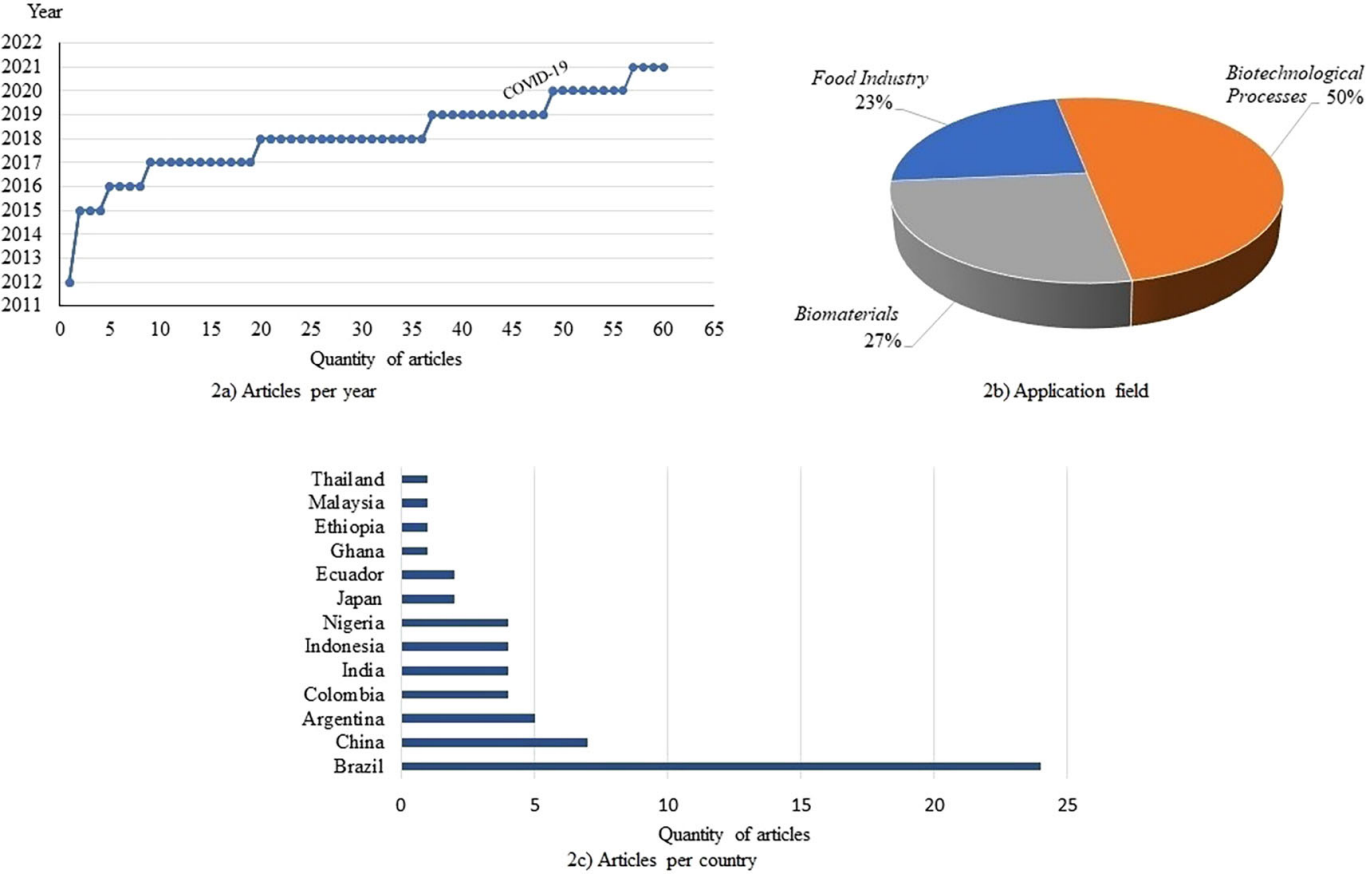
Study statistics on the use of cassava bagasse: a) Articles per year; b) Application field; c) Articles per country.

**Figure 3.**
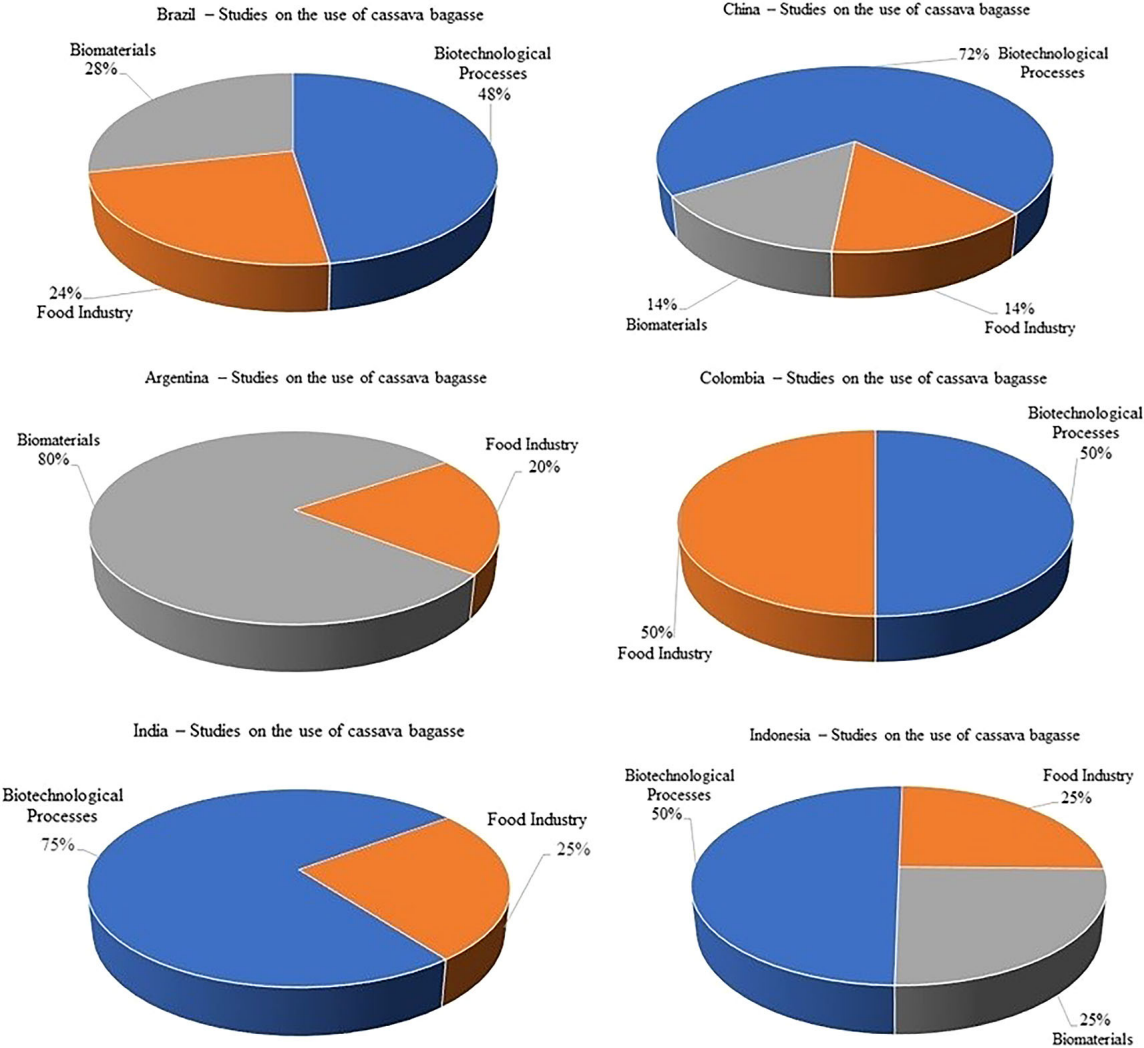
Field of research application of cassava bagasse by country.

The studies reported in the scientific literature relate to use of cassava bagasse from 2012–2021 ( Figure 2a). They show a clear interest in this research topic by the scientific community, who tend to seek alternatives for the use of cassava bagasse, particularly in three study fields ( Figure 2b). Fifty percent of the studies correspond to the use of cassava bagasse in the biotechnological processes field: fungi production, organic acids, xanthan gum, bio-pigments, and production of biofuels (bioethanol, biomethane, hydrogen, and butanol); 23% of the studies are oriented to cassava bagasse use in the animal and human food industry: balanced feed, gastronomic diets, cookie making, ice cream, and others; and 27% of the studies are oriented towards use of cassava bagasse in the production of biofilms and other biomaterials as biodegradable reinforcement material, foam production, and nanofibers, and others. This study trend from 2012–2021 is marked by Brazil as the country contributing more than 39% of the research in this field of study, followed by China with 13%, and Argentina, Colombia, India, Indonesia and Nigeria with percentages between 6 and 7% ( Figure 2c). Figure 3 illustrates the interest field of countries leading the studies on the use of cassava bagasse. In these illustrations it can be seen that South America and Asia show the greatest interest in biotechnological processes and development of biomaterials.

Advances in the different options for the use of cassava bagasse highlight the need to simultaneously advance in efficient technologies for the drying of this waste. For effective use of post-harvest cassava bagasse, prior drying is essential ( Rodríguez *et al*., 2016). Drying is widely used in the food industry to extend shelf life of products by inhibiting growth of microorganisms and enzyme activity. Traditional drying techniques such as convective drying have drawbacks, mainly because they require a long processing time and generate changes in products’ properties ( Prosapio & Norton, 2017). Some pre-drying treatments have been proposed with good results for the drying of fruits and vegetables. For example, Table 3 describes some of the studies that examine the emerging technologies of ultrasound and electrical pulses in combination with osmotic dehydration and convective drying operations of various agroindustrial products. These technologies are useful for agribusiness that is always looking for innovative technologies to improve the efficiency of its processes and reduce energy consumption.

**Table 3.** Technological applications of the combined techniques of ultrasound, electrical pulses, dehydration and drying of different products.

Author, year	Combined techniques	Application
Bozkir *et al*. (2019); Correa *et al*. (2017); Allahdad *et al*. (2018); Li *et al*. (2020)	Ultrasound - Osmotic Dehydration - Drying	Fruits, tubers (yams).
Prosapio & Norton (2017)	Osmotic dehydration - Drying	Fruits.
Rodriguez *et al*. (2019); Souza *et al*. (2019); La Fuente *et al*. (2017); Zhang & Abatzoglou (2019); Fan *et al*. (2017); Kowalski & Rybicki (2017); Ricce *et al*. (2016); Vera *et al*. (2018); Asrofi *et al*. (2018); Abral *et al*. (2017); Kowalski (2015); Li *et al*. (2018); Musielak *et al*. (2016); Vieira *et al*. (2018); Ortuño *et al*. (2010); Fan *et al*. (2017); Fernandes *et al*. (2008)	Ultrasound - Drying	Fruits and vegetables, food, biocomposites and porous materials.
Prithani & Dash (2020); Rahaman *et al*. (2019); Sakooei-Vayghan *et al*. (2019); Sharma & Dash (2019); Kiani *et al*. (2018); Barman & Badwaik (2017); Nowacka *et al*. (2018); Goula *et al*. (2017); Feng *et al*. (2018)	Ultrasound Osmotic Dehydration	Fruits, tubers (yam, cassava, potato, sweet potato), bulbs.
Dermesonlouoglou *et al*. (2018)	Electrical pulses Osmotic dehydration - Drying	Fruits
Yu *et al*. (2018); Traffano-Schiffo *et al*. (2016); Yildiz *et al*. (2016); Semenoglou *et al*. (2020)	Electrical pulses Osmotic dehydration.	Fruits and Fillets
Wiktor *et al*. (2019)	Ultrasound - Electrical pulses - Drying	Vegetables
Dehnad *et al*. (2016); Moreno-Vilet *et al.* (2018); Troy *et al*. (2016); Hernández *et al*. (2019); Ahmed *et al.* (2016); Ciurzynska *et al*. (2016); Ramya & Jain (2017)	Emerging Technologies and Osmotic Dehydration	General applications in various products

In this context, more studies are required that examine the use of environmentally friendly techniques to lower energy use and the environmental footprint induced by food processing and waste generation throughout the supply chain. This will allow the replacement of traditional techniques with more efficient and sustainable processes ( López-Pedrouso *et al*., 2019). In this regard, osmotic dehydration has received considerable attention in recent years as it is one of the simplest and cheapest dehydration treatments that reduces energy consumption and accelerates drying time ( Feng *et al*., 2018). This has led to the evaluation of different osmotic agents for the dehydration of fruits and tubers as a pretreatment to convective drying in order to improve energy efficiency and process costs (Abbasi *et al*., cited by Sharma and Dash, 2019). When osmotic dehydration is assisted by the emerging technologies of electric pulses, high hydrostatic pressure, ultrasound, centrifugal force, vacuum, and gamma irradiation, it has been shown to improve the dehydration process by increasing cell membrane permeability and generating a greater diffusion and mass transfer rate. These combined operations have been shown to reduce drying time, and energy costs associated with this operation ( Ahmed *et al*., 2016; Dehnad *et al*., 2016).

These novel or emerging technologies arose to meet the need to improve conventional drying technologies. Today, they are the subject of study alone or in combination with conventional techniques to improve product safety, shelf life and processing time ( Moreno-Vilet *et al*., 2018). Table 3 shows articles that apply these technologies individually or in combination, but with other purposes rather than improving the efficiency of the osmotic dehydration and convective drying processes that use air as a drying agent. Studies excluded are those that apply these combined techniques in operations of lyophilization, freezing, frying, homogenization, extraction of bioactive compounds, extraction of essential oils, extraction of phenolic compounds, protein extraction, crystallization operations, recovery extraction of intracellular compounds, inactivation of microorganisms, and enzyme inactivation.

Other alternative technologies, not object of this study, that have been shown to improve food processing and preservation conditions are supercritical fluids, subcritical water extraction, microwave-assisted treatments, and infrared radiation ( Gamboa-Santos *et al*., 2016).

Emerging technologies can be generally used as drivers of osmotic dehydration efficiency and convective drying in various agroindustrial products. Use of emerging technologies of ultrasound and electrical pulses has been successfully applied to improve the efficiency of osmotic dehydration and drying processes of fruits, vegetables, meat, and tubers such as yams, sweet potatoes, cassava, and potatoes, as well as in some polymeric matrices This generates changes in their structure and makes them more permeable with the consequent reduction in drying time of up to 50% and 70% in some cases depending on their structure, degree of hardness and porosity ( Bozkir *et al.,* 2019; Prithani & Dash, 2020; Rahaman *et al*., 2019; Sakooei-Vayghan *et al*., 2019; Sharma & Dash, 2019; Li *et al*., 2020; Allahdad *et al*., 2018; Feng *et al*., 2018; Goula *et al*., 2017; Yu *et al*., 2018; Dermesonlouoglou *et al*., 2018; Barman & Badwaik, 2017; Traffano-Schiffo *et al*., 2016; Yildiz *et al*., 2016).

**Figure 4.**
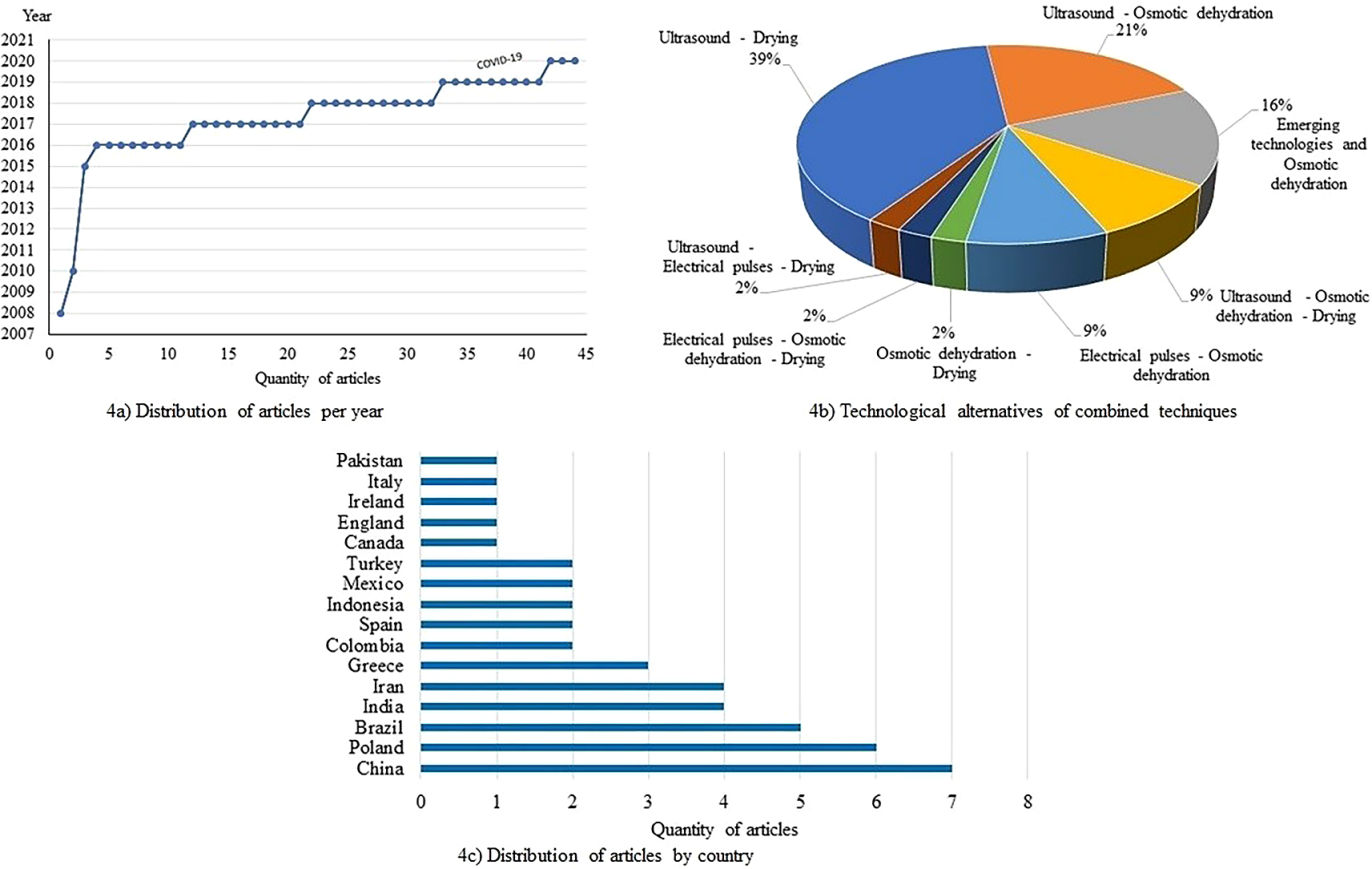
Statistics of studies on the applications of the combined techniques of ultrasound, electrical pulses, dehydration and drying of different products: a) Distribution of articles per year; b) Technological alternatives of combined techniques; c) Distribution of articles by country.

Figure 4 presents the statistics of the application of combined ultrasound techniques, electrical pulses, dehydration, and drying of different products from 2008–2021. Use of emerging technologies in the agro-food industry for the dehydration and drying of fruits, vegetables, and tubers, shows a trend in studies, especially since 2016 ( Figure 4a). Figure 4b shows that the major investigations have focused on studying the combined use of ultrasound technologies with convective drying and osmotic dehydration. In other studies, use of electrical pulses with osmotic dehydration has been reported. For countries such as China, Poland, Brazil, India, Iran, and Greece ( Figure 4c). This demonstrates an open research agenda for studies using emerging technologies alone or in combination in food dehydration and drying processes.

The study of dehydration and drying of fruits and vegetables, among other products, in the period between 2010–2021 has been framed by the use of techniques combined with the assistance of emerging technologies, resulting in improved energy efficiency and lower the operating cost of the drying process. These findings, added to the volume of research related to drying of fruits and vegetables in recent years, highlight the trend of studies on dehydration prior to conventional drying of various products, mainly fruits and vegetables, supported in turn by emerging technologies.

## Discussion

The high humidity of cassava bagasse makes it a perishable product in which all kinds of microorganisms proliferate, generating decomposition by uncontrolled fermentation, causing bad odors, affecting both, processing plants and surrounding regions, and becoming an environmental problem that must be properly managed. The aforementioned, together with the large volumes of generated waste, have drawn the attention of the scientific community, especially in the last five years. Studies have aimed at finding alternatives for the use of cassava bagasse, which have been orientated towards three main areas: 1) Use in the food industry, 2) Biotechnological applications to generate higher value-added products, and 3) Use as reinforcement material in biomaterials. Nevertheless, the main problem encountered during the management of high-humidity waste such as cassava bagasse is related to handling, storage, transportation, and conservation, a problem further increased due to the daily generation of high production volumes of this residue. So, it is essential that this waste goes through a pre-treatment to eliminate moisture thus reducing its mass and volume to favor handling and preserve the product. In this sense, it is necessary to look for new dehydration or drying alternatives, opening a research agenda aimed at evaluating the application of new technologies or emerging technologies or combinations of these with traditional technologies, which lead to an efficient drying process of cassava bagasse, for exploitation purposes, and promote the cassava starch industry towards a sustainable agroindustry.

The vast range of alternatives reported in the scientific literature on the use of cassava bagasse and its specific use options in different applications, justify the need to advance in technological innovation research in efficient dehydration and/or drying processes of this agro-industrial by-product. In this regard, it was found that use of emerging ultrasound and electrical pulse technologies in the agrifood industry favor mass and heat transfer processes in osmotic dehydration and drying operations, being a trend of studies especially as of 2016. Despite the fact that these combined techniques have been efficiently applied in osmotic dehydration and drying processes of various products, such as fruits, vegetables, fillets, and tubers including yams, sweet potatoes and potatoes, as well as in some polymer matrices, there is no scientific literature that reports how these technologies sways the efficiency of the osmotic dehydration process and subsequent drying of cassava bagasse and other agro-industrial residues. In this sense, one of the limitations of the scoping review process in this article was to consolidate the search strategy for cassava bagasse, due to the different names that cassava ( *Manihot esculenta*) can have. It is known in different countries with different local names including cassava, aipim, lumu, tapioca, guacamota, casabe or mandioca. In addition, various names are adopted in each country for the residue: cassava bagasse, cassava bran, typyraty or cassava starch bagasse. Therefore, we may not have been able to retrieve all relevant articles on cassava bagasse. Furthermore, by restricting our search to journal articles in English, we may have also excluded relevant research in languages other than English.

## Conclusions

Efficient management of agroindustrial waste to lower its negative environmental externality is among the important challenges for a sustainable industry. Negative externalities generated by the cassava bagasse waste in starch-producing plants have attracted the attention of the scientific community to seek alternative solutions aimed at the sustainable use of this waste in three large areas: biotechnological, food, and biomaterials. Nonetheless, internationally today, the strategies implemented by the cassava starch industry have not been able to relieve the negative externality largely generated by the humidity held in this agroindustrial waste, making it unmanageable and unaffordable for post-industrial purposes. In this sense, technological alternatives are necessary for the efficient drying of cassava bagasse.

Even though studies have been reported that provide alternatives for the use of cassava bagasse, these studies leave unsolved the daily accumulation of large volumes of this residue in the cassava starch plants with the consequent problems of management and environmental pollution. As a result, it is not only important to continue advancing in studies on the use of this residue, but it is also necessary to advance in studies aimed at achieving an efficient drying of this residue that allows its handling and subsequent use and drives the cassava starch industry towards a sustainable agribusiness. In this sense, in recent years, the application of combined techniques of emerging and traditional technologies have been used successfully to achieve efficient dehydration of various products, also becoming an alternative for its application in the efficient drying of cassava bagasse and other agro-industrial residues, especially using those technologies that, due to their simplicity and economy, make their evaluation interesting in these processes.

With this regard, we have found that osmotic dehydration with the assistance of ultrasound technologies and electrical pulses as a pretreatment for drying fruits, vegetables, and tubers, marks a trend in studies with excellent results in terms of reducing drying time and energy expenditure. This has become an interesting option to be evaluated in the efficiency of drying agro-industrial residues of husks and bagasse among other residues from the extractive food industry that carry a high-water content. Accordingly, it is paramount to review the use of these technologies in supporting the drying of cassava bagasse.

The follow-up to this article presents a review of the current state of osmotic dehydration technology, alone or assisted by the emerging technologies of ultrasound or electric pulses, applied as a pretreatment to improve the efficiency of the drying operation in various agroindustrial products. The aspects addressed are the effects on the drying process, the related factors or parameters and the operating conditions, the main results, the equipment used, and the products to which this type of combined technology has been applied. The information in Part II of this article provides elements for future research, which may lead to the application of these combined technologies to improve the efficiency of the osmotic dehydration process and drying of new products, by-products, and agroindustrial waste. The findings in Part II will be useful as parameters for the design of the drying process for agroindustrial waste with high moisture content.

## Data availability

### Underlying data

All data underlying the results are available as part of the article and no additional source data are required.

### Reporting guidelines

Open Science Framework: PRISMA checklist and flow chart for ‘Post-industrial context of cassava bagasse and trend of studies towards a sustainable industry: A scoping review – Part I’. https://doi.org/10.17605/OSF.IO/RQTGC ( Serpa-Fajardo *et al*., 2022).

Data are available under the terms of the Creative Commons Zero “No rights reserved” data waiver (CC0 1.0 Public domain dedication).
