# Supplementary material for: Post-industrial context of cassava bagasse and trend of studies towards a sustainable industry: A scoping review – Part I
Source: F1000Res. 2022 May 23;11:562. [Version 1] doi: 10.12688/f1000research.110429.1 (PMC9772581; doi:10.12688/f1000research.110429.1)
Supplement: Peer review history

# Peer review: Reviewer response for version 1

**DOI:** 10.5256/f1000research.122031.r152452

**Published:** 2022-12-21

**Authors:** Fernando Salazar Arrieta

**Affiliations:** 1 Universidad Escuela Naval de Cadetes Almirante Padilla, Cartagena, Colombia

It is an interesting and current topic in the post-pandemic reality. It represents an interesting analysis for companies and the new way in which they are called to operate in the business and business context, given the current circumstances of uncertainty and more so in the field of agribusiness and by-products.

 The methodological development of the article complies with the theme, from the approach to the problem, the approach to the solution, the analysis of the results and the conclusions.

 The topic is topical, which guarantees an additional contribution or analysis that particularly contributes to the productive sector once the Pandemic has been overcome, which has led to a global crisis and local repercussions. For these reasons I have given the final concept that it be indexed with minor adjustments in the part indicated in the evaluation.

 From here it is important to consider that new studies arise based on strengthening the productive sector of small and medium enterprises that suffer most of the impacts.

 As additional comments, I wish to expand the reasons why I consider that the article represents a topic of interest to be indexed. Both the topic addressed and the object of study, which are small and medium-sized companies, make this article work as a basis or source of analysis for these companies. Also, it presents interesting elements to be considered by companies and incorporated into their management or administration plans in the current post-pandemic circumstances, where high levels of uncertainty flood the reality of most or all of the world's countries and economies, which suggests elements so that companies see them as tools that allow them to operate in the markets, trying not to be affected to a greater extent, assigns to the article that I have reviewed the conditions and characteristics of a work of interest to the academic community and to the productive sector, even more supportive.

 From the methodological point of view, the article meets the standards of the Journal and scientific rigor for its indexing, evidenced from the problem, materials and methods, conclusions and references for the investigation.

Are the rationale for, and objectives of, the Systematic Review clearly stated?

Yes

Is the statistical analysis and its interpretation appropriate?

Yes

Are sufficient details of the methods and analysis provided to allow replication by others?

Yes

Are the conclusions drawn adequately supported by the results presented in the review?

Yes

Reviewer Expertise:

Humanitarian logistics; agrifood security, strategic planning logistics, financial logistics, reverse logistics.

I confirm that I have read this submission and believe that I have an appropriate level of expertise to confirm that it is of an acceptable scientific standard.

# Peer review: Reviewer response for version 1

**DOI:** 10.5256/f1000research.122031.r139181

**Published:** 2022-09-16

**Authors:** Eduardo Martínez-Mendoza

**Affiliations:** 1 Industrial engineering, Universidad del Istmo, Tehuantepec, Mexico

This work is important because studied an actual problem, and the authors have justified it within the work. The relevance of this work is that identifies technologies that could be applied to improve waste cassava bagasse management. PRISMA method is appropriate for this work. The research contributes to proposing using current technological solutions on vegetables or fruit to reduce cassava bagasse externalities.

 The paper developed correctly the PRISMA methodology and has reviewed vast and recent decade literature from important databases, that they have classified and categorized adequately.

 The results are relevant and systematically presented according to the research method.

Are the rationale for, and objectives of, the Systematic Review clearly stated?

Yes

Is the statistical analysis and its interpretation appropriate?

Yes

Are sufficient details of the methods and analysis provided to allow replication by others?

Yes

Are the conclusions drawn adequately supported by the results presented in the review?

Yes

Reviewer Expertise:

Sustainable development, Complex Systems

I confirm that I have read this submission and believe that I have an appropriate level of expertise to confirm that it is of an acceptable scientific standard.
